# Enhancement of transcription efficiency by TAR-Tat system increases the functional expression of human olfactory receptors

**DOI:** 10.1371/journal.pone.0306029

**Published:** 2024-06-25

**Authors:** Ichie Ojiro, Hibiki Katsuyama, Ryusei Kaneko, Satoshi Ogasawara, Takeshi Murata, Yuko Terada, Keisuke Ito

**Affiliations:** 1 Department of Food and Nutritional Sciences, Graduate School of Integrated Pharmaceutical and Nutritional Sciences, University of Shizuoka, Shizuoka, Japan; 2 Department of Chemistry, Graduate School of Science, Chiba University, Chiba, Japan; 3 School of Food and Nutritional Sciences, University of Shizuoka, Shizuoka, Japan; Nuclear Science and Technology Research Institute, ISLAMIC REPUBLIC OF IRAN

## Abstract

Humans have approximately 400 different olfactory receptors (hORs) and recognize odorants through the repertoire of hOR responses. Although the cell surface expression of hORs is critical to evaluate their response, hORs are poorly expressed on the surface of heterologous cells. To address this problem, previous studies have focused on hOR transportation to the membrane. Nevertheless, the response pattern of hORs to odorants has yet to be successfully linked, and the response sensitivity still remains to be improved. In this study, we demonstrate that increasing the transcriptional level can result in a significant increase in cell surface and functional expression of hORs. We used the TAR-Tat system, which increases the transcription efficiency through positive feedback, and found that OR1A1, OR6N2, and OR51M1 exhibited robust expression. Moreover, this system induces enhanced hOR responses to odorants, thus defining four hORs as novel n-hexanal receptors and n-hexanal is an inverse agonist to one of them. Our results suggested that using the TAR-Tat system and increasing the transcriptional level of hORs can help understanding the relationship between hORs and odorants that were previously undetectable. This finding could facilitate the understanding of the sense of smell by decoding the repertoire of hOR responses.

## Introduction

Humans can recognize more than one trillion types of smell through the response patterns of approximately 400 olfactory receptors (hORs) [1–3]. Therefore, evaluating the response pattern of hORs for each smell can help analyzing in detail the sense of smell based on the human olfactory perception mechanism [4].

The response pattern of hORs to odorants can be evaluated using the hOR assay, which is performed in heterologous cell systems and requires the cell surface expression of the hOR [5]. Nevertheless, several hORs exhibit poor expression on the surface of heterologous cells, a problem considered a bottleneck in the sensitivity of the hOR assay system [6]. In 2003, it was reported that mouse ORs (mI7 and mOREG) accumulate in the ER during their translocation to the plasma membrane [7]. Subsequently, several studies have focused on hOR membrane translocation to increase the cell surface expression of hORs, and several strategies have been demonstrated to be effective in transferring ORs to the cell surface [8]. For instance, adding the first 20 amino acids of rhodopsin on the N-terminal of mouse ORs (mORs) could enhance the cell surface expression of mI7 and I-C6 [9]. Furthermore, addition of the Lucy tag and the IL-6–HaloTag^®^ could enhance the cell surface expression of several mORs and hOR [5, 10]. RTP1S, RTP1 and 2, and REEP1 were also identified as chaperone proteins that facilitate the trafficking of hORs to the cell surface [3, 11, 12]. Moreover, coexpression with other GPCRs was found to enhance the cell surface expression of mORs (M71 and M72) [13].

On the basis of these previous studies, the assay system that involves the coexpression of RTP1S, G_αolf_, and Ric-8b (enhancers of the signal of G_αolf_) with Rho-tagged hORs has been widely used [5]. Some ligands were identified for several hORs using this system [11, 14]. However, the cell surface expression levels of several hORs still remain low [8]. Considering the sensitivity of human olfactory perception, only odorants with a reasonably high concentration or hORs with a strong response can be evaluated. Hence, the overall pattern of the hOR response to an odor has not been elucidated. In fact, the majority of hORs remain orphan receptors, with no known ligand even when chaperone proteins or Rho N-terminal additions are applied [5]. Furthermore, it has recently been demonstrated that odor information is represented by the map of not only the “activated” but also the “inhibitory” response [15]. Analysis of the hOR response pattern has been more highlighted in the field of science and industry than before [16], and a more sensitive OR assay system is desired. To achieve this goal, a novel strategy is required in addition to those used in previous studies. In this study, we focused on “gene expression,” which is a step before membrane translocation and has never received sufficient focus, and attempted to improve the cell surface expression of hORs by increasing the transcription level of *hOR* gene.

## Methods

### Plasmid construction

*hOR* genes were synthesized by Eurofins Japan and subcloned into a simple mammalian expression vector, pBApo-CMV neo vector (Takara Bio), which has the CMV promoter containing Lucy-(N-AGACCCCAGATCCTGCTGCTCCTGGCCCTGCTGACCCTAGGCCTGGCT-C), FLAG-(N-GACTACAAAGACGATGACGACAAG-C), and Rho-(N-AATGGCACAGAAGGCCCTAACTTCTACGTGCCCTTCTCCAATGCGACGGGTGTGGTACGC-C) tags. The UniProt ID of olfactory receptor genes used for analysis were listed in supplemental data (S1 Table). To enhance the transcriptional level of hORs, we used the transcriptional activation TAR-Tat system based on HIV-1 [17]. In the TAR-Tat system, the positive feedback of Tat binding to TAR increases the transcription of the target protein [18]. To use the TAR-Tat system, hORs and other tags were amplified using Prime STAR Max (Takara Bio) and subcloned into pHEK293 Ultra Expression Vector I (Takara Bio), which contains the TAR-Tat system, by Gibson assembly. The sequence of the cloned receptors was confirmed by sequencing (Eurofins Japan). For each experiment, the hOR in the pBApo-CMV neo vector (hOR/pBApo) was used as a control and compared with the hOR in pHEK293 Ultra Expression Vector I (hOR/pHEK).

### Cell culture

Flp-In T-REx-293 cells stably expressing accessory proteins (RTP1S, G_αolf_, and Ric8-b) were established using the Flp-In T-REx system [19, 20]. Accessory proteins-expressing cells were maintained in high-glucose Dulbecco’s modified Eagle medium (Sigma-Aldrich) containing 10% fetal bovine serum (Biowest) and 1% antibiotic–antimycotic (Nacalai tesque) at 37°C with 5% CO_2_.

### Real-time RT-PCR

Accessory proteins-expressing cells (7.0 × 10^5^ cells/well) were seeded in a 35-mm dish 20 h before transfection in high-glucose Dulbecco’s modified Eagle medium containing 10% fetal bovine serum. Lipofectamine 2000 (Thermo Fisher Scientific) (5 μL/well) was used for the transfection of hOR/pBApo and hOR/pHEK. A total of 1.0 μg of hOR DNA was transfected per dish. At 28 h posttransfection, total RNA was extracted using RNAiso Plus (Takara Bio) according to the manufacturer’s protocol. cDNA was synthesized using ReverTra Ace^®^ qPCR RT Master Mix with gDNA Remover (TOYOBO) according to the manufacturer’s protocol. For real-time RT-PCR, primer (forward: TGAACGGGAAGCTCACTGG, reverse: TCCACCACCCTGTTGCTGTA) was used for GAPDH, and primers (forward: GTCTTGGGTGATTGGAAATGC, reverse: CTGGTTGCCACAGAAGGACA), (forward: ATTGGGGCTGTGCTGAAGAT, reverse: AGCAAGTGTTCGGTCAAGGG) and (forward: ATGATGTCCTTTGACCGCCT, reverse: TGGGAGAGGACCACAGATCC) were used for OR1A1 and OR6N2 and OR51M1, respectively. In a 96-well plate, 400 ng of cDNA was mixed with 10 μL of PowerUp SYBR Green Master Mix (Thermo Fisher Scientific) and 4 μL of primer mix (10 μM). Fluorescence was measured and analyzed using the Thermal Cycler Dice^®^ Real Time System (Takara Bio). The mRNA levels of hORs were calculated using the ΔCt method relative to those of GAPDH. The amplification efficiency of all genes to be analyzed was almost 1, indicating a 2-fold difference in concentration if there was a difference of one cycle of PCR amplification (Ct value) between the two samples. Therefore, the expression levels of the target genes were compared by substituting values into the formula (2^−ΔCt^) in the analysis.

### HiBiT cell surface expression assay

The cell surface expression level of hORs was quantified using the Nano-Glo® HiBiT Extracellular Detection System (Promega), which consists of the HiBiT peptide and the LgBiT protein. The LgBiT is impermeable and hence can access only the hOR, which has the HiBiT tag and expresses on the cell surface. The hORs containing the Lucy tag followed by the HiBiT tag, the FLAG tag and Rho tag were generated by PCR using primer (forward: GTGAGCGGCTGGCGGCTGTTCAAGAAGATTAGCGACTACAAAGACGATGACGACA, reverse: GCTAATCTTCTTGAACAGCCGCCAGCCGCTCACAGCGGCCGCAGCCAGGCCTAGG). The PCR program was set at 98°C for 1 min, followed by 35 cycles of denaturation at 98°C for 10 s, annealing at 55°C for 30 sec, extension at 72°C for 10 s, followed by a final extension at 72°C for 2 min. The transfection of the hOR plasmid with (hOR/pHEK) and without (hOR/pBApo) the TAR-Tat system to the accessory proteins-expressing cells (2.0 × 10^4^ cells/well) was performed in black 96-well tissue culture plates containing 50 ng of each construct using Lipofectamine 2000 (Thermo Fisher Scientific) (400 nL/well). After 28 h, the plates were equilibrated to room temperature, and the Nano-Glo HiBiT extracellular reagent (Promega) was added to each well (100 μL/well), according to to the manufacturer’s protocol. After 10 min of incubation with the reagent at room temperature, luminescence signals were measured using GloMax (Promega). Cells (2.0 × 10^4^ cells/well) that were transfected by the pBApo-CMV neo vector and pHEK293 Ultra Expression Vector I were used as the negative control. All luminescence values were subtracted by the L_mock_ value and normalized (ΔRLU).

### HiBiT expression assay

The expression level of hORs in the cells was quantified using the Nano-Glo® HiBiT Lytic Detection System (Promega), which consists of two subunits, the 11-amino HiBiT peptide and the LgBiT protein, that can interact with the HiBiT tag to reconstitute the bright, luminescent enzyme. The lytic reagent was used to disrupt cells so that impermeable LgBiT can access HiBiT-tagged hORs. The hORs containing the Lucy tag followed by the HiBiT tag, the FLAG tag and Rho tag were generated and transfected in the same way as "HiBiT cell surface expression assay". After 28 h, the plates were equilibrated to room temperature, and the 100 μL Nano-Glo® HiBiT Lytic Reagent (Promega) was added and mixed with cells by pipetting, according to the manufacturer’s protocol. After 10 min of incubation with the reagent at room temperature, luminescence signals were measured using GloMax (Promega). Cells that were transfected by the pBApo-CMV neo vector and pHEK293 Ultra Expression Vector I were used as the negative control. All luminescence values were subtracted by the L_mock_ value and normalized (ΔRLU).

### hOR assay and data analysis

The GloSensor™ Technology (Promega) was used for the luciferase assay of the hOR response. As the negative control, cells that were transfected by the pBApo-CMV neo vector and pHEK293 Ultra Expression Vector I were used as mock cells. hOR/pHEK and hOR/pBApo (50 ng/well) were transfected into the accessory proteins-expressing cell line (40000 cells/well) using Lipofectamine 2000 (Thermo Fisher Scientific) (400 nL/well) along with pGlosensor™-22F cAMP biosensor plasmid (Promega) (50 ng/well) in a 96-well plate. At 28 h posttransfection, the cells were washed with an assay buffer (140 mM NaCl, 10 mM HEPES, 5 mM KCl, 1 mM CaCl_2_・2H_2_O, 10 mM d-glucose, pH 7.5) and replaced with the assay buffer (50 μL/well) containing 2% GloSensor cAMP substrate and preincubated for 1 h at 25°C. Then, the assay buffer was added (130 μL/well). After 10 min of equilibration, the luminescence was measured (L_0_). Odorants diluted in 10% DMSO/assay buffer were treated (20 μL/well). After 9 min, the luminescence was measured (L_9min_). First, all the L_9min_ values were subtracted by the L_0_ value (ΔL) and divided by the L_0_ value (ΔL/L_0_). The concentration range of the odorants was different for each odorant, but the minimum was 0.01 μM and the maximum was 3 mM. For all hORs, luciferase assays with hOR/pBApo and hOR/pHEK were conducted in the same 96-well plate each time, and luminescence was normalized to a maximum value of 100. Dose–response curve images were graphed using the Graph Pad Prism 9 software (Graph Pad Software).

### Comprehensive analysis of hORs against n-hexanal

Although 396 types of olfactory receptors have been detected in the human genome [2], the hORs that have a stop codon in the middle of the expected sequence or do not function were excluded from this study. Transfection was performed as described in the “hOR assay,” but 12.5 ng of 379 hOR plasmids (hOR/pBApo and hOR/pHEK) was transfected with the 12.5 ng pGlosensor^TM^-22F cAMP biosensor plasmid (Promega) in 384 plates per well. After loading the GloSensor cAMP substrate and preincubation, the assay buffer was added (30 μL/well). After 10 min of equilibration, luminescence was measured (L_0_). Then, 50 mM n-hexanal, which was diluted with 10% DMSO/assay buffer, was added (4.7 μL/well). After 9 min, luminescence was measured (L_9min_). All the L_9min_ values were subtracted by the L_0_ value (ΔL).

## Results

### Effect of the TAR-Tat system on the hOR gene transcription level

**Fig 1:**
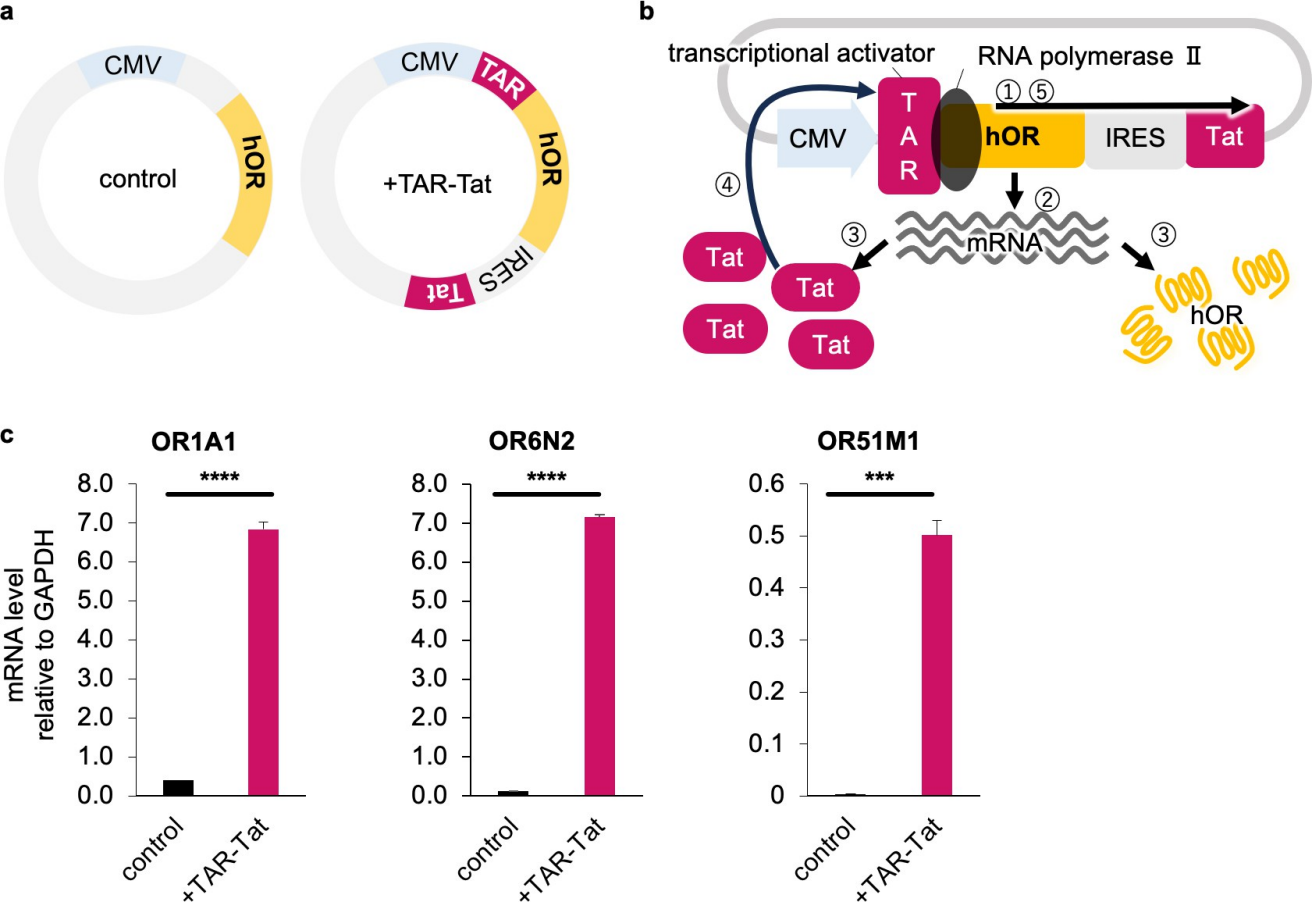
mRNA level of hORs with or without the TAR-Tat system. (a) The design of the control plasmid and the +TAR-Tat plasmid were used in this study. The plasmid, which has a CMV promoter and encodes *hOR*, was used as a control (pBApo-CMV neo vector). With the TAR-Tat system, the TAR sequence is incorporated in the 5ʹ untranslated region and the Tat sequence is downstream of the hOR sequence (pHEK293 Ultra Expression Vector I). (b) The principle of the TAR-Tat system. The Tat, transcribed and expressed with the hOR, binds to the TAR in the untranslated region. Then, the activated TAR phosphorylates RNA polymerase II and results in the promotion of transcription. (c) The mRNA level of OR1A1/6N2/51M1 was measured by real-time RT-PCR and analyzed by ΔCt analysis. Data are expressed as average (n = 3) ± SE (error bars). ***P < 0.005 and ****P < 0.001, unpaired t-*test*, for comparison of control and with the TAR-Tat system.

The vector with the TAR-Tat system was used to increase the level of hOR transcription (Fig 1A). The TAR-Tat system is based on the principle that when Tat binds to TAR in HIV-LTR, RNA polymerase II is phosphorylated and activates transcription (Fig 1B [21]. After Tat, which has been transcribed and translated together with the target protein, binds to TAR in the 5ʹ untranslated region, transcription is promoted. We chose OR1A1, a well-studied hOR [6], and OR6N2 and OR51M1 to represent diverse lineages within the hOR repertoire to investigate the effect of the TAR-Tat system on transcription. We found that the mRNA levels of OR1A1, OR6N2, and OR51M1 were higher by 17-, 52-, and 137-fold, respectively, compared the TAR-Tat system to no TAR-Tat system (Fig 1C). Although the level of transcription differed among these three hORs, they all increased when the TAR-Tat system was used. The sequence identity of *OR1A1* and *OR51M1* was 46.9%, and that of *OR6N2* and *OR51M1* was 46.5%. More than 90% of hORs showed >45% sequence identity. This result suggests that the TAR-Tat system can be adapted to a wide range of hORs.

### Effect of the TAR-Tat system on the hOR cell surface expression

In previous studies, the influence of altering the N-terminal of the hORs toward the hORs response assay system has been reported. To investigate the impact of the HiBiT tag on hORs function, HiBiT-tagged OR1A1 and OR1A1 were transfected into accessory protein-expressing cells, and their response to (+)-carvone was evaluated. As in the case without the HiBiT tag, a dose-dependent response was detected with the HiBiT tag (S1 Fig). This result indicates that adding the HiBiT tag does not seriously influence the OR1A1 function. Before measuring the cell surface expression level of hORs, we evaluated whether LgBiT specifically binds to the HiBiT tag attached to the N-terminus of ORs and whether it exhibits luminescence. HiBiT tests have been performed on mock cells and HiBiT-tagged OR1A1 expression cells. Robust luminescence values were demonstrated when the OR1A1 with the HiBiT sequence was inserted in both vectors, viz., the pBApo-CMV neo vector and the pHEK293 Ultra Expression Vector I (S2 Fig). Based on these results, we determined that the HiBiT test can be used to detect the cell surface expression of hORs.

**Fig 2:**
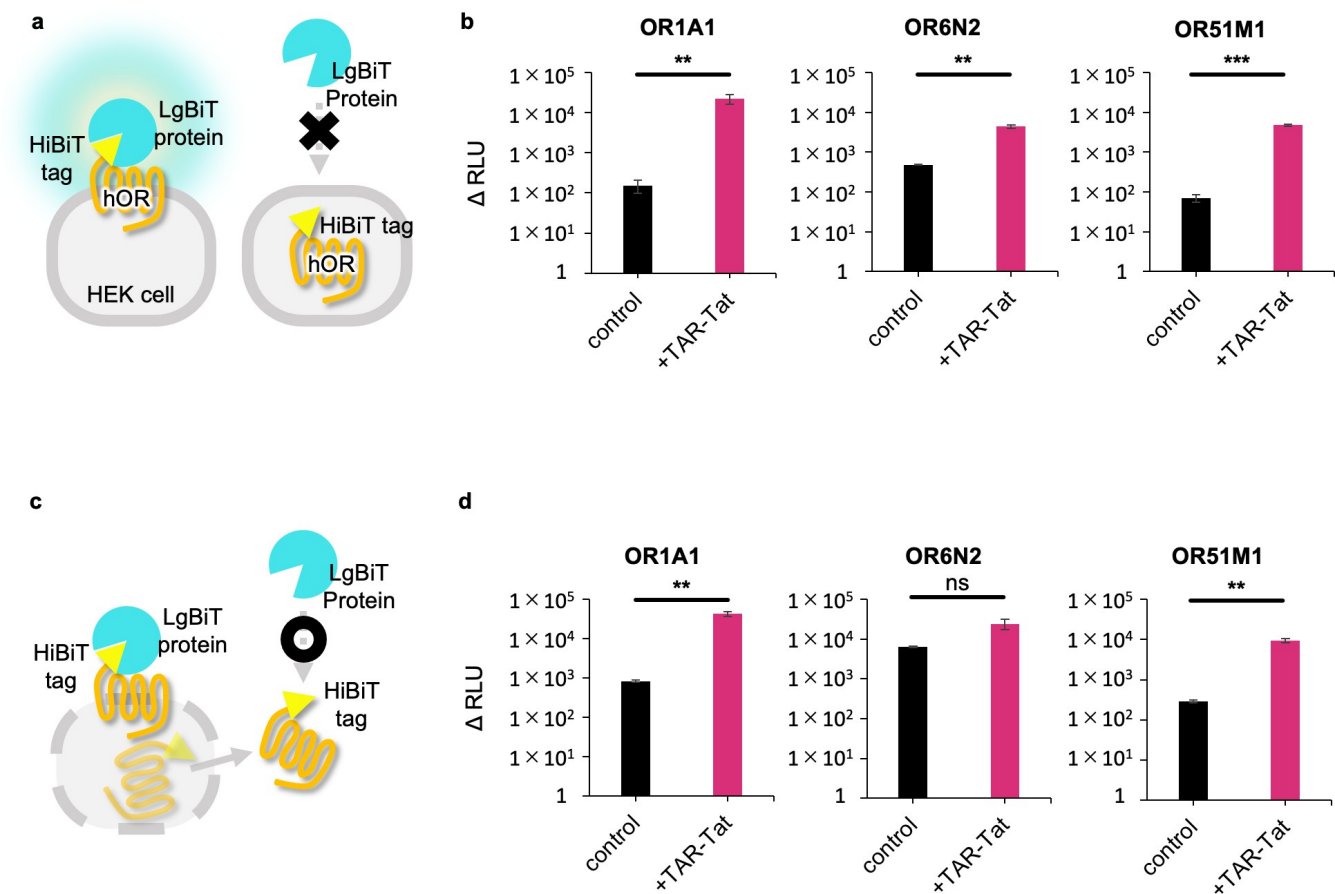
Expression level of hORs with or without the TAR-Tat system. (a) Principle of the HiBiT cell surface expression assay. Nonlytic detection reagent containing the substrate furimazine and LgBiT protein binds to hORs that express on the cell surface. LgBiT is membrane-impermeable and cannot bind to intracellular hORs. (b) Luminescence signals of OR1A1/6N2/51M1 expressed on the cell surface. Data are expressed as average (n = 4) ± SE (error bars). (c) Principle of the HiBiT expression assay. Lytic reagent disrupts cells, and the substrate furimazine and LgBiT protein can bind to hORs in the cells. (d) Luminescence signals of OR1A1/6N2/51M1 expressed in cell lysates. Data are expressed as average (n = 4) ± SE (error bars). **P < 0.01 and ***P < 0.005, unpaired t-*test*, for comparison of control and with the TAR-Tat system.

To check whether increased hOR transcription causes increased cell surface expression of hOR in heterogeneous cells, we evaluated the surface and overall expression levels of OR1A1, OR6N2, and OR51M1 using the HiBiT system (Fig 2). In the control data (without the TAR-Tat system), the luminescence value of OR6N2 was higher than that of OR1A1, suggesting that OR6N2 is more easily expressed on the cell surface than OR1A1. Each type of hOR is known to have different cell surface expression levels; however, the cause of this difference still remains unknown [8]. Interestingly, when the TAR-Tat system was used, similar high expression levels were observed for OR1A1, OR6N2, and OR51M1. With the TAR-Tat system, the expression levels of OR1A1, OR6N2, and OR51M1 were 147-, 9-, and 67-fold higher than those without the TAR-Tat system, respectively. These data suggest that the TAR-Tat system can significantly increase the cell surface expression of hORs that have low or regular surface expression. Moreover, the overall expression levels of OR1A1 and OR51M1 significantly increased with the TAR-Tat system (Fig 2). In the case of OR6N2, there were no significant differences, however, it showed a trend in the same direction as the other hORs. These results indicate that the TAR-Tat system can raise the whole hOR expression, leading to an increase in the expression of the cell surface. These findings suggest that the amplified transcription of hORs using the TAR-Tat system is effective for improving cell surface expression and overcoming the bottleneck in the OR expression system.

### Effect of the TAR-Tat system on the sensitivity of the hOR assay system

**Fig 3:**
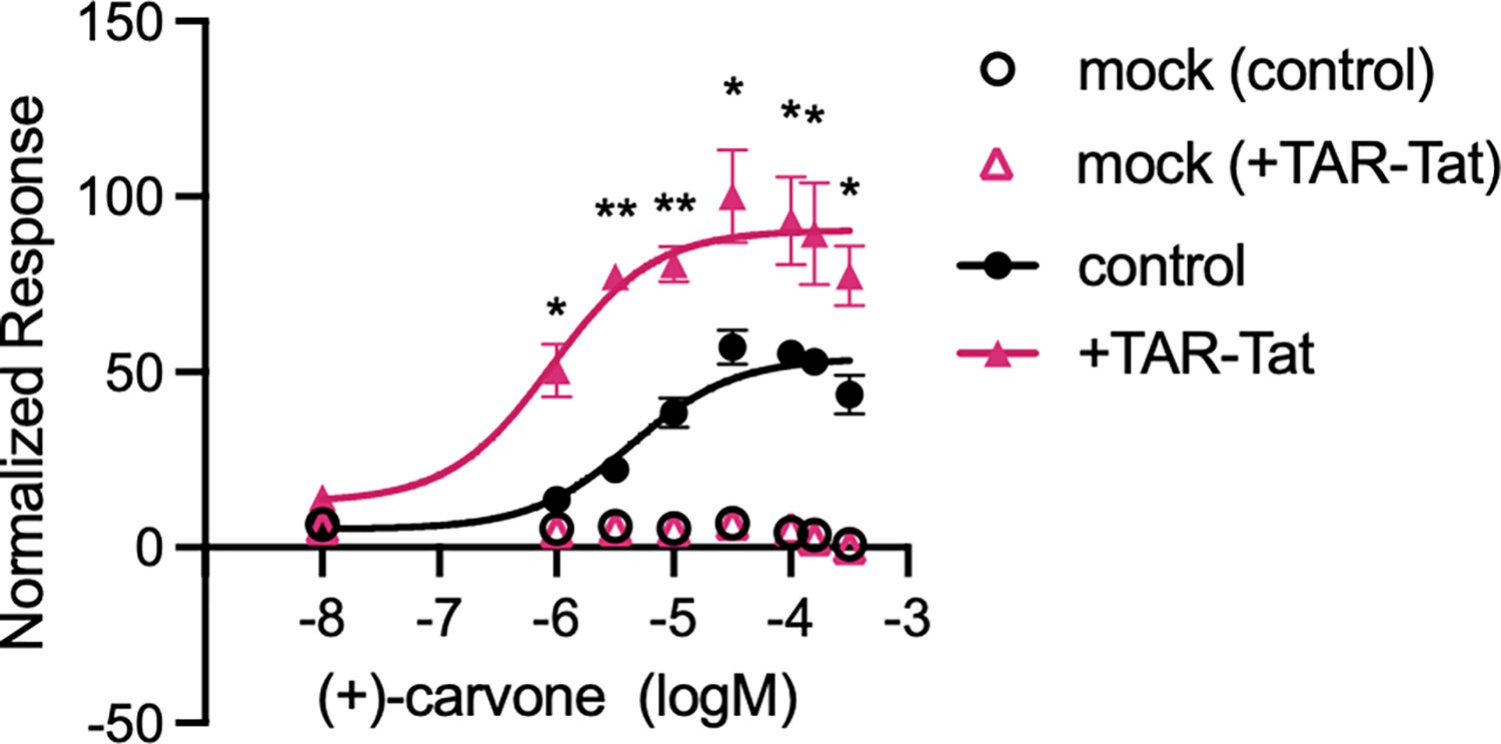
Dose–response curves of OR1A1 to (+)-carvone with or without the TAR-Tat system. The responses of OR1A1 against (+)-carvone were evaluated using the control plasmid (OR1A1/pBApo) or the plasmid having the TAR-Tat system (OR1A1/pHEK). As a negative control, pBApo-CMV neo vector and pHEK Ultra Expression Vector I were used for mock cells. The luminescence was normalized to a maximum value of 100. The y-axis denotes the normalized response, and the x-axis represents the concentration of (+)-carvone. Data are expressed as average (n = 3) ± SD (error bars). *P < 0.05 and **P < 0.005, unpaired t-*test*, for comparison of control and with the TAR-Tat system.

To examine the effect of the TAR-Tat system on the hOR assay system, we measured the response of OR1A1 against (+)-carvone with or without the TAR-Tat system (Fig 3). According to the result, the top value of OR1A1 dose–response curve to (+)-carvone was 53.9 without the TAR-Tat system, whereas it was 90.4 with the TAR-Tat system. The EC_50_ value also shifted from 4.4 μM to 905 nM with the TAR-Tat system. A study demonstrating that the coexpression of G_αolf_ in the mouse OR assay increased the response sensitivity also reported the same increase in the maximum value and decrease in EC_50_ value as observed in the present study [22]. These data suggest that using the TAR-Tat system could enhance the sensitivity of the hOR assay system.

**Fig 4:**
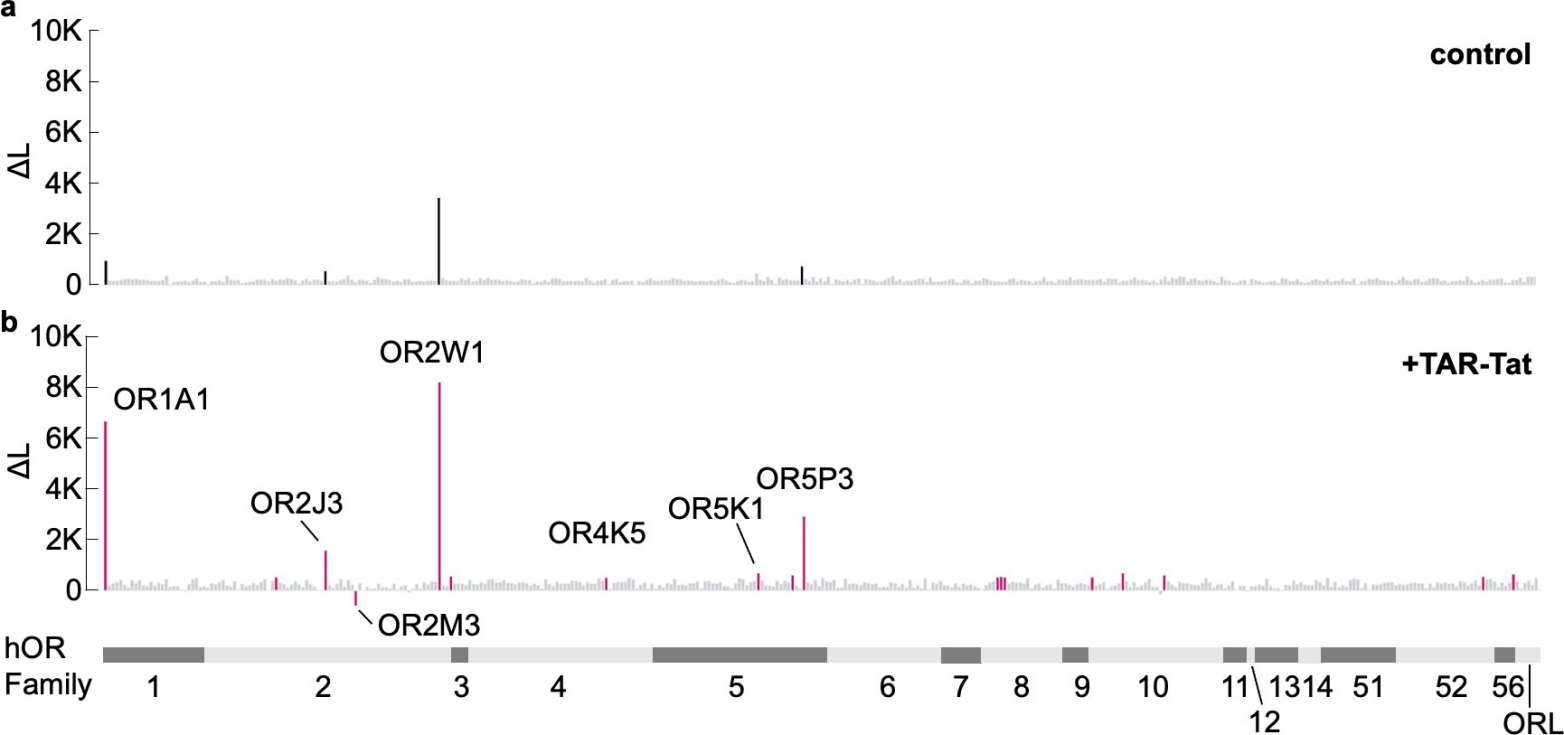
Screening of n-hexanal receptors from the hOR repertoire with or without the TAR-Tat system. The responses of 379 hORs against 500 μM n-hexanal were comprehensively measured using the control plasmid (hORs/BApo) (a) or the plasmid with the TAR-Tat system (hORs/pHEK) (b) (n = 1). Those with ΔL >500 or <−500 are shown in black for the control and magenta for the TAR-Tat system, and all others are shown in gray. X-axis bars represent OR families.

To confirm the improvement in the sensitivity of the hOR assay system with the implementation of the TAR-Tat system, we comprehensively analyzed the hOR response using n-hexanal as a model with or without the TAR-Tat system and then compared the type of hORs detected and the response values (Fig 4). Adapting the TAR-Tat system led to significant changes in several responses of hORs. Without the TAR-Tat system, the response values (ΔL) of OR1A1, OR2J3, OR2W1, and OR5P3 were 974, 549, 3463, and 740, respectively, but with the TAR-Tat system, the respective values were 6675, 1580, 9310, and 2931, showing a dramatic increase. Interestingly, the response value (ΔL) of OR2M3 was negative (−620) only in the hOR assay system with the TAR-Tat system. ΔL values of >500 were shown by 4 hORs (OR1A1, OR2J3, OR2W1, and OR5P3) without the TAR-Tat system and 17 hORs (OR1A1, OR2B3, OR2J3, OR2W1, OR2Z1, OR4K5, OR5K1, OR5M3, OR5P3, OR8B3, OR8B4, OR8B8, OR10A2, OR10C1, OR10H3, OR52N4, and OR56B1) with the TAR-Tat system.

**Fig 5:**
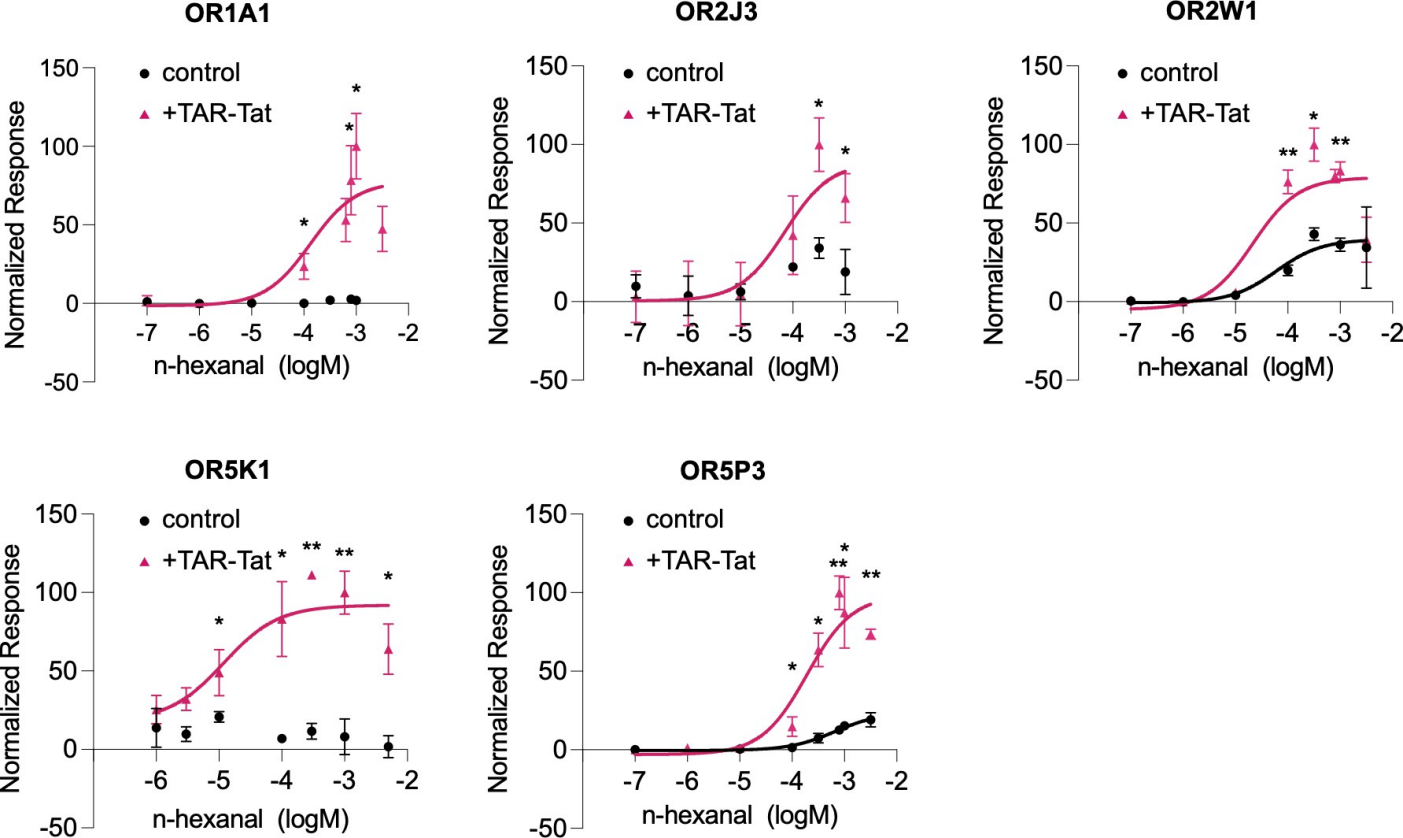
Dose–response curves of hORs to n-hexanal with or without the TAR-Tat system. The dose-responses of hORs that show responses in the screening against n-hexanal were measured using the control plasmid (hOR/pBApo) or the plasmid with the TAR-Tat system (hOR/pHEK). Luminescence was normalized to a maximum value of 100. The y-axis denotes the normalized response, and the x-axis represents the concentration of (+)-carvone. The fitting curves were shown if R^2^ were above 0.7. Data are expressed as average (n = 3) ± SD (error bars). *P < 0.05 and **P < 0.005, unpaired t-*test*, for comparison of control and with the TAR-Tat system.

We next evaluated the response to n-hexanal individually for each of the 17 hORs, which showed a ΔL of >500, in conditions with and without the TAR-Tat system, to determine whether a concentration-dependent response existed. We found no concentration-dependent response for OR2B3, OR2Z1, OR4K5, OR5M3, OR8B3, OR8B4, OR8B8, OR10A2, OR10C1, OR10H3, OR52N4, and OR56B1 with or without the TAR-Tat system (S2 Table). Concentration-dependent responses were observed for OR2W1 and OR5P3 with and without the TAR-Tat system (Fig 5). The EC_50_ value for each hOR was as follows: OR2W1/pBApo, 21 μM; OR2W1/pHEK, 60 μM; OR5P3/pBApo, 191 μM; OR5P3/pHEK, 672 μM (S2 Table). For OR2W1, the EC_50_ value of n-hexanal (7.9 μM) has been reported in a previous study [11], and it is comparable to the value calculated in this study. There was no significant change in EC_50_ values depending on the presence of the TAR-Tat system in other hORs (S2 Table). However, the maximum values increased by 2.0-fold for OR2W1 and 4.1-fold for OR5P3 (Fig 5). For OR1A1, OR2J3, and OR5K1, there were concentration-dependent responses only with the TAR-Tat system, and the EC_50_ values were as follows: OR1A1, 995 μM; OR2J3, 71 μM; OR5K1: 11 μM (Fig 5) (S2 Table).

**Fig 6:**
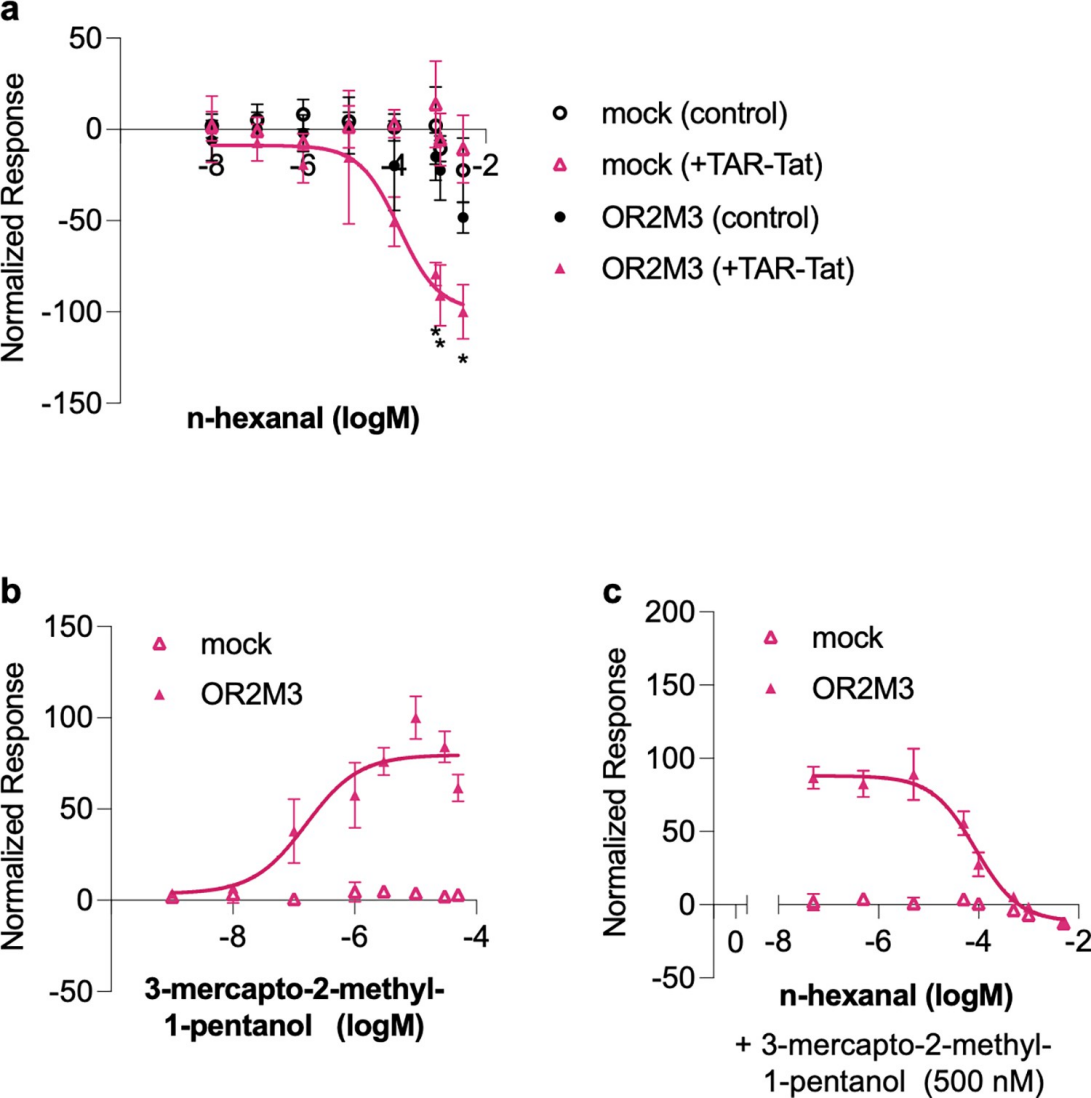
Inhibitory response of OR2M3 against n-hexanal. (a) The inhibitory effects of n-hexanal on the constitutive activity of OR2M3. Luminescence was normalized to a minimum value of −100. (b) Dose–response curves of OR2M3 to 3-mercapto-2-methyl-1-pentanol using OR2M3/pHEK plasmid. Luminescence was normalized to a maximum value of 100. (c) Dose–response curves of the inhibitory response of OR2M3 against n-hexanal in the presence of agonist (500 nM 3-mercapto-2-methyl-1-pentanol) using OR2M3/pHEK plasmid. 100 indicate the maximum response value of OR2M3 for 3-mercapto-2-methyl-1-pentanol. Data are expressed as average (n = 3) ± SD (error bars). *P < 0.05 and **P < 0.005, unpaired t-*test*, for comparison of control and with the TAR-Tat system.

Moreover, when OR2M3, which showed significant suppression, was analyzed individually, the luminescence values decreased in an n-hexanal concentration-dependent manner only in the case with the TAR-Tat system (Fig 6A). Recent research has clearly shown that hORs exhibit not only active but also inhibitory responses to odorants, and inverse agonists, which can inhibit GPCRs with constitutive activity [23], have also been reported [15]. Therefore, we evaluated whether n-hexanal suppresses the agonist response of OR2M3. We first evaluated the response of OR2M3 against 3-mercapto-2-methyl-1-pentanol, a known agonist of OR2M3, and found a concentration-dependent response similar to the results of previous studies [24], with the EC_50_ value being 159 nM (Fig 6B). Then, we coadministered 500 μM 3-mercapto-2-methyl-1-pentanol and different concentrations of n-hexanal and found that the active responses of OR2M3 to 3-mercapto-2-methyl-1-pentanol were suppressed in an n-hexanal concentration-dependent manner (Fig 6C). These findings indicate that n-hexanal inhibits OR2M3 as an inverse agonist.

## Discussion

The continued process of decoding the response pattern of hORs has been significantly hampered by the inability to express hORs in heterologous cells [25]. Previous studies on the hOR assay system have generally focused on hOR transportation to cell membranes. In this study, we took a different approach by focusing on the “gene expression” process before membrane transfer and attempted to improve the cell surface expression level of hORs. The increased transcription of hORs by the TAR-Tat system significantly increased the cell surface expression of hORs (Fig 2) and increased the sensitivity of the hOR assay system (Fig 3). This resulted in the discovery of three novel receptors and inverse agonists responsive to n-hexanal (Figs 5 and 6).

Following a study that reported considerable accumulation of hORs in the ER as detected by immunostaining, it is believed that ER stress, which is caused by protein accumulation, is the bottleneck for the cell surface expression of hORs [26]. Therefore, there has been extensive research to improve the trafficking of hORs from the ER to the cell surface [12, 26]. The addition of Rho and Lucy tags to the N-terminal sequence of hORs and the coexpression of the chaperone protein RTP1S, which have been reported to promote membrane migration in previous studies, were also adapted to the expression system in the present study. Although there is a possibility that ER stress was enhanced by the TAR-Tat system, the increased response of OR1A1 and other hORs suggested that ER stress was not high enough to prevent detection of the response. This study therefore demonstrated that increasing the “transcription level” can be an efficient method to overcome the difficulty associated with hOR expression in addition to previous approaches.

Although the TAR-Tat system was used in this study, other systems can also improve transcription. For instance, it has been reported that codon optimization can increase the mRNA level of protein using the mite allergen Der f 7 as a model protein in *Aspergillus oryzae* [27]. Moreover, although the CMV promoter was used in this study, other promoters, such as EF-1α and CAG, which have been confirmed as higher expression promoters than the CMV promoter in studies using GFP proteins [28], can be candidates to enhance the transcription level of hORs. Furthermore, WPRE (woodchuck hepatitis virus posttranscriptional regulatory element) in the pcDNA™3.4 TOPO™ TA Cloning Kit (Thermo Fisher) increases mRNA stability and ultimately increases the transcription of the target gene [29, 30]. The cell surface expression of hORs could be further enhanced by attempting other methods of increasing transcription or by combining them.

The cell surface expression of GPCRs is essential for their functional analysis using heterologous cells. For instance, Tan et al. optimized N-terminal signal sequences to increase the cell surface expression of the bitter taste receptor (TAS2R) and succeeded in improving the sensitivity of the TAS2R assay system [31]. Similar to the TAS2R study, this study elucidated that increasing the cell surface expression of hOR is effective in detecting the clear responses of hORs. Moreover, OR1A1, OR2J3 and OR5K1 were identified as novel receptors that respond to n-hexanal, and their EC_50_ values were calculated. These data reaffirm that increasing the cell surface expression of hOR can improve the sensitivity of the hOR assay system, which is consistent with previous studies [5, 12].

In this study, we used n-hexanal as a model for screening. n-hexanal is the major off-flavor compound of the smell of soybeans. Therefore, the response receptors identified in this study may be targets in the search for masking, such as in the field of plant-based meat development. OR2W1 is the only hOR that was reported to be activated by n-hexanal with known EC_50_ values from previous studies [11]. However, as odors are formed by the response pattern of hORs, more effective masking can be anticipated by analyzing not only the hORs that exhibit strong responses but also the composition of the hORs that comprise the pattern. For instance, the screening results revealed that n-hexanal acts as an inverse agonist of OR2M3. Hence, the agonist of OR2M3, such as 3-mercapto-2-methyl-1-pentanol, can change the pattern of n-hexanal hOR responses and function as a masking reagent. Hence, this system, which can now detect hOR responses that could not be detected using the conventional assay system, is effective for designing odors using hORs as an indicator. Furthermore, if EC_50_ values can be calculated, the composition of the hORs that change with the concentration of the odorant compound can also be ascertained. The EC_50_ values could not be calculated for some hORs using the conventional assay system without the TAR-Tat system, because the response values detected were low and concentration-dependent responses could not be detected (Fig 5) (S2 Table). Recently, studies on the ligand selectivity of hORs by deep learning have also progressed [32–34]. Because the EC_50_ value is a quantitative indicator of the ligand effect of a compound, calculating the EC_50_ value would be useful for such AI analysis.

Using the TAR-Tat system, we successfully identified n-hexanal as an inverse agonist of OR2M3 (Fig 6). A wide range of inhibitory responses against constitutive activity have been reported to occur in olfactory sensory neurons, demonstrating that inhibitory responses occur at the receptor level [35, 36]. Such inhibitory responses also contribute to the receptor response pattern. Nevertheless, as a highly sensitive assay system capable of detecting sufficient activity is required to detect receptors with inhibitory responses, there has been limited knowledge about inverse agonists or antagonists of hORs [37]. The hOR assay system developed in this study, which can also detect inhibition against constitutive activity, may be immensely useful for data acquisition on hOR inverse agonists and response patterns.

To summarize, the hOR assay system becomes more sensitive with the implementation of the TAR-Tat system, resulting in the successful identification of novel active and inhibitory response receptors. In the future, this advanced assay system may be used to be used to elucidate the relationship between hORs and odorants.

## Supporting information

S1 Table UniProt ID for human odorant receptors used in this paper. (DOCX)

S2 Table EC_50_ values (μM) of n-hexanal concentration–response relationship for hOR/pBApo and hOR/pHEK. The term "n.d." stands for "not detected".
(DOCX)

S1 Fig Dose–response curves of OR1A1 to (+)-carvone with or without the HiBiT tag. The responses of OR1A1 against (+)-carvone were evaluated with or without the HiBiT tag. The luminescence was normalized to a maximum value of 100. The y-axis denotes the normalized response, and the x-axis represents the concentration of (+)-carvone. Data are expressed as average (n = 3) ± SD (error bars).
(TIF)

S2 Fig Luminescence of HiBiT tagged OR1A1. (TIF)
