# Supplementary material for: Enhancement of transcription efficiency by TAR-Tat system increases the functional expression of human olfactory receptors
Source: PLoS One. 2024 Jun 25;19(6):e0306029. doi: 10.1371/journal.pone.0306029 (PMC11198769; doi:10.1371/journal.pone.0306029)
Supplement: S1 Table — (DOCX) [file pone.0306029.s001.docx]

Supporting information

S1 Table

| **Name of the gene** | **Uniprot ID** | **Name of the gene** | **Uniprot ID** | **Name of the gene** | **Uniprot ID** |
| --- | --- | --- | --- | --- | --- |
| OR1A1 | Q9P1Q5 | OR4F6 | Q8NGB9 | OR8K3 | Q8NH51 |
| OR1A2 | Q9Y585 | OR4K1 | Q8NGD4 | OR8K5 | Q8NH50 |
| OR1B1 | Q8NGR6 | OR4K13 | Q8NH42 | OR8S1 | Q8NH09 |
| OR1C1 | Q15619 | OR4K14 | Q8NGD5 | OR8U1 | Q8NH10 |
| OR1D2 | P34982 | OR4K15 | Q8NH41 | OR9A4 | Q8NGU2 |
| OR1D5 | P58170 | OR4K17 | Q8NGC6 | OR9G1 | Q8NH87 |
| OR1E1 | P30953 | OR4K2 | Q8NGD2 | OR9G4 | Q8NGQ1 |
| OR1E2 | P47887 | OR4K5 | Q8NGD3 | OR9I1 | Q8NGQ6 |
| OR1F1 | O43749 | OR4L1 | Q8NH43 | OR9K2 | Q8NGE7 |
| OR1F12 | Q8NHA8 | OR4M1 | Q8NGD0 | OR9Q1 | Q8NGQ5 |
| OR1G1 | Q9TU86 | OR4M2 | Q8NGB6 | OR9Q2 | Q8NGE9 |
| OR1I1 | O60431 | OR4N2 | Q8NGD1 | OR10A2 | Q9H208 |
| OR1J1 | Q8NGS3 | OR4N4 | Q8N0Y3 | OR10A3 | P58181 |
| OR1J2 | Q8NGS2 | OR4N5 | Q8IXE1 | OR10A4 | Q9H209 |
| OR1J4 | Q8NGS1 | OR4P4 | Q8NGL7 | OR10A5 | Q9H207 |
| OR1K1 | Q8NGR3 | OR4Q3 | Q8NH05 | OR10A6 | Q8NH74 |
| OR1L1 | Q8NH94 | OR4S1 | Q8NGB4 | OR10A7 | Q8NGE5 |
| OR1L3 | Q8NH93 | OR4S2 | Q8NH73 | OR10AD1 | Q8NGE0 |
| OR1L4 | Q8NGR5 | OR4X1 | Q8NH49 | OR10AG1 | Q8NH19 |
| OR1L6 | Q8NGR2 | OR4X2 | Q8NGF9 | OR10C1 | Q96KK4 |
| OR1L8 | Q8NGR8 | OR5A1 | Q8NGJ0 | OR10D3 | Q8NH80 |
| OR1M1 | Q8NGA1 | OR5A2 | Q8NGI9 | OR10G2 | Q8NGC3 |
| OR1N1 | Q8NGS0 | OR5AC2 | Q9NZP5 | OR10G3 | Q8NGC4 |
| OR1N2 | Q8NGR9 | OR5AK2 | Q8NH90 | OR10G4 | Q8NGN3 |
| OR1Q1 | Q15612 | OR5AN1 | Q8NGI8 | OR10G6 | Q8NH81 |
| OR1S1 | Q8NH92 | OR5AP2 | Q8NGF4 | OR10G7 | Q8NGN6 |
| OR1S2 | Q8NGQ3 | OR5AR1 | Q8NGP9 | OR10G8 | Q8NGN5 |
| OR2A1 | Q8NGT9 | OR5AS1 | Q8N127 | OR10G9 | Q8NGN4 |
| OR2A12 | Q8NGT7 | OR5AU1 | Q8NGC0 | OR10H1 | Q9Y4A9 |
| OR2A14 | Q96R47 | OR5B12 | Q96R08 | OR10H2 | O60403 |
| OR2A2 | Q6IF42 | OR5B17 | Q8NGF7 | OR10H3 | O60404 |
| OR2A25 | A4D2G3 | OR5B2 | Q96R09 | OR10H4 | Q8NGA5 |
| OR2A4 | O95047 | OR5B21 | A6NL26 | OR10H5 | Q8NGA6 |
| OR2A42 | Q8NGT9 | OR5B3 | Q8NH48 | OR10J1 | P30954 |
| OR2A5 | Q96R48 | OR5C1 | Q8NGR4 | OR10J5 | Q8NHC4 |
| OR2A7 | Q96R45 | OR5D13 | Q8NGL4 | OR10K1 | Q8NGX5 |
| OR2AE1 | Q8NHA4 | OR5D14 | Q8NGL3 | OR10K2 | Q6IF99 |
| OR2AG1 | Q9H205 | OR5D16 | Q8NGK9 | OR10P1 | Q8NGE3 |
| OR2AG2 | A6NM03 | OR5D18 | Q8NGL1 | OR10Q1 | Q8NGQ4 |
| OR2AJ1(ORL4524) | Q8NGZ0 | OR5F1 | O95221 | OR10R2 | Q8NGX6 |
| OR2AK2 | Q8NG84 | OR5H1 | A6NKK0 | OR10S1 | Q8NGN2 |
| OR2AP1 | Q8NGE2 | OR5H14 | A6NHG9 | OR10T2 | Q8NGX3 |
| OR2AT4 | A6N04 | OR5H15 | A60H6 | OR10V1 | Q8NGI7 |
| OR2B11 | Q5JQS5 | OR5H2 | Q8NGV7 | OR10W1 | Q8NGF6 |
| OR2B2 | Q9GZK3 | OR5H6 | Q8NGV6 | OR10X1 | Q8NGY0 |
| OR2B3 | O76000 | OR5I1 | Q13606 | OR10Z1 | Q8NGY1 |
| OR2B6 | P58173 | OR5J2 | Q8NH18 | OR11A1 | Q9GZK7 |
| OR2C1 | O95371 | OR5K1 | Q8NHB7 | OR11G2 | Q8NGC1 |
| OR2C3 | Q8N628 | OR5K2 | Q8NHB8 | OR11H1 | Q8NG94 |
| OR2D2 | Q9H210 | OR5K3 | A6NET4 | OR11H2 | Q8NH07 |
| OR2D3 | Q8NGH3 | OR5K4 | A6NMS3 | OR11H4 | Q8NGC9 |
| OR2F1 | Q13607 | OR5L1 | Q8NGL2 | OR11H6 | Q8NGC7 |
| OR2F2 | O95006 | OR5L2 | Q8NGL0 | OR11L1 | Q8NGX0 |
| OR2G2 | Q8NGZ5 | OR5M1 | Q8NGP8 | OR12D2 | P58182 |
| OR2G3 | Q8NGZ4 | OR5M10 | Q6IEU7 | OR12D3 | Q9UGF7 |
| OR2H1 | Q9GZK4 | OR5M11 | Q96RB7 | OR13A1 | Q8NGR1 |
| OR2H2 | O95918 | OR5M3 | Q8NGP4 | OR13C2 | Q8NGS9 |
| OR2J2 | O76002 | OR5M8 | Q8NGP6 | OR13C3 | Q8NGS6 |
| OR2J3 | O76001 | OR5M9 | Q8NGP3 | OR13C4 | Q8NGS5 |
| OR2K2 | Q8NGT1 | OR5P3 | Q8WZ94 | OR13C5 | Q8NGS8 |
| OR2L13 | Q8N349 | OR5R1 | Q8NH85 | OR13C8 | Q8NGS7 |
| OR2L2 | Q8NH16 | OR5T1 | Q8NG75 | OR13C9 | Q8NGT0 |
| OR2L3 | Q8NG85 | OR5T2 | Q8NGG2 | OR13D1 | Q8NGV5 |
| OR2L5 | Q8NG80 | OR5T3 | Q8NGG3 | OR13F1 | Q8NGS4 |
| OR2L8 | Q8NGY9 | OR5V1 | Q9UGF6 | OR13H1 | Q8NG92 |
| OR2M2 | Q96R28 | OR5W2 | Q8NH69 | OR13J1 | Q8NGT2 |
| OR2M3 | Q8NG83 | OR6A2 | O95222 | OR14A16 | Q8NHC5 |
| OR2M4 | Q96R27 | OR6B1 | O95007 | OR14A2 | Q96R54 |
| OR2M5 | A3KFT3 | OR6B2 | Q6IFH4 | OR14C36 | Q8NHC7 |
| OR2M7 | Q8NG81 | OR6B3 | Q8NGW1 | OR14I1 | A6048 |
| OR2S2 | Q9NQN1 | OR6C1 | Q96RD1 | OR14J1 | Q9UGF5 |
| OR2T1 | O43869 | OR6C2 | Q9NZP2 | OR14K1 | Q8NGZ2 |
| OR2T10 | Q8NGZ9 | OR6C3 | Q9NZP0 | OR51A2 | Q8NGJ7 |
| OR2T11 | Q8NH01 | OR6C4 | Q8NGE1 | OR51A4 | Q8NGJ6 |
| OR2T12 | Q8NG77 | OR6C6 | A6NF89 | OR51A7 | Q8NH64 |
| OR2T2 | Q6IF00 | OR6C65 | A6NJZ3 | OR51B2 | Q9Y5P1 |
| OR2T27 | Q8NH04 | OR6C68 | A60L8 | OR51B4 | Q9Y5P0 |
| OR2T29 | Q8NH02 | OR6C70 | A6NIJ9 | OR51B5 | Q9H339 |
| OR2T3 | Q8NH03 | OR6C74 | A6NCV1 | OR51B6 | Q9H340 |
| OR2T33 | Q8NG76 | OR6C75 | A6NL08 | OR51D1 | Q8NGF3 |
| OR2T34 | Q8NGX1 | OR6C76 | A6NM76 | OR51E1 | Q8TCB6 |
| OR2T35 | Q8NGX2 | OR6F1 | Q8NGZ6 | OR51E2 | Q9H255 |
| OR2T4 | Q8NH00 | OR6J1 | Q8NGC5 | OR51F1 | A6NGY5 |
| OR2T5 | Q6IEZ7 | OR6K2 | Q8NGY2 | OR51F2 | Q8NH61 |
| OR2T6 | Q8NHC8 | OR6K3 | Q8NGY3 | OR51G1 | Q8NGK1 |
| OR2T8 | A6NH00 | OR6K6 | Q8NGW6 | OR51G2 | Q8NGK0 |
| OR2V1 | Q8NHB1 | OR6M1 | Q8NGM8 | OR51I1 | Q9H343 |
| OR2V2 | Q96R30 | OR6N1 | Q8NGY5 | OR51I2 | Q9H344 |
| OR2W1 | Q9Y3N9 | OR6N2 | Q8NGY6 | OR51L1 | Q8NGJ5 |
| OR2W3 | Q7Z3T1 | OR6P1 | Q8NGX9 | OR51M1 | Q9H341 |
| OR2Y1 | Q8NGV0 | OR6Q1 | Q8NGQ2 | OR51Q1 | Q8NH59 |
| OR2Z1 | Q8NG97 | OR6S1 | Q8NH40 | OR51S1 | Q8NGJ8 |
| OR3A1 | P47881 | OR6T1 | Q8NGN1 | OR51T1 | Q8NGJ9 |
| OR3A2 | P47893 | OR6V1 | Q8N148 | OR52A1 | Q9UKL2 |
| OR3A3 | P47888 | OR6X1 | Q8NH79 | OR52A5 | Q9H2C5 |
| OR3A4 | P47883 | OR6Y1 | Q8NGX8 | OR52B2 | Q96RD2 |
| OR4A15 | Q8NGL6 | OR7A10 | O76100 | OR52B4 | Q8NGK2 |
| OR4A16 | Q8NH70 | OR7A17 | O14581 | OR52B6 | Q8NGF0 |
| OR4A47 | Q6IF82 | OR7A5 | Q15622 | OR52D1 | Q9H346 |
| OR4A5 | Q8NH83 | OR7C1 | O76099 | OR52E2 | Q8NGJ4 |
| OR4B1 | Q8NGF8 | OR7C2 | O60412 | OR52E4 | Q8NGH9 |
| OR4C11 | Q6IEV9 | OR7D2 | Q96RA2 | OR52E5 | Q8NH55 |
| OR4C12 | Q96R67 | OR7D4 | Q8NG98 | OR52E6 | Q96RD3 |
| OR4C13 | Q8NGP0 | OR7E24 | Q6IFN5 | OR52E8 | Q6IFG1 |
| OR4C15 | Q8NGM1 | OR7G1 | Q8NGA0 | OR52H1 | Q8NGJ2 |
| OR4C16 | Q8NGL9 | OR7G2 | Q8NG99 | OR52I1 | Q8NGK6 |
| OR4C3 | Q8NH37 | OR7G3 | Q8NG95 | OR52I2 | Q8NH67 |
| OR4C46 | A6NHA9 | OR8A1 | Q8NGG7 | OR52J3 | Q8NH60 |
| OR4C5 | Q8NGB2 | OR8B12 | Q8NGG6 | OR52K1 | Q8NGK4 |
| OR4C6 | Q8NH72 | OR8B2 | Q96RD0 | OR52K2 | Q8NGK3 |
| OR4D1 | Q15615 | OR8B3 | Q8NGG8 | OR52L1 | Q8NGH7 |
| OR4D10 | Q8NGI6 | OR8B4 | Q96RC9 | OR52M1 | Q8NGK5 |
| OR4D11 | Q8NGI4 | OR8B8 | Q15620 | OR52N1 | Q8NH53 |
| OR4D2 | P58180 | OR8D1 | Q8WZ84 | OR52N2 | Q8NGI0 |
| OR4D5 | Q8NGN0 | OR8D2 | Q9GZM6 | OR52N4 | Q8NGI2 |
| OR4D6 | Q8NGJ1 | OR8D4 | Q8NGM9 | OR52N5 | Q8NH56 |
| OR4D9 | Q8NGE8 | OR8G5 | Q8NG78 | OR52R1 | Q8NGF1 |
| OR4E2 | Q7TQQ0 | OR8H1 | Q8NGG4 | OR52W1 | Q6IF63 |
| OR4F15 | Q8NGB8 | OR8H2 | Q8N162 | OR56A1 | Q8NGH5 |
| OR4F16 | Q6IEY1 | OR8H3 | Q8N146 | OR56A3 | Q8NH54 |
| OR4F17 | Q8NGA8 | OR8I2 | Q8N0Y5 | OR56A4 | Q8NGH8 |
| OR4F21 | O95013 | OR8J1 | Q8NGP2 | OR56A5 | P0C7T3 |
| OR4F29 | Q6IEY1 | OR8J3 | Q8NGG0 | OR56B1 | Q8NGI3 |
| OR4F4 | Q96R69 | OR8K1 | Q8NGG5 | OR56B4 | Q8NH76 |
| OR4F5 | Q8NH21 |  |  | ORL3007 | Q8NHC6 |

| **Name of the gene** | **PMC ID** |
| --- | --- |
| ORL4502 | PMC218742 |
| ORL4674 | PMC218742 |
| ORL4696 | PMC218742 |
| ORL4698 | PMC218742 |
| ORL4699 | PMC218742 |
