# Supplementary material for: Enhancement of transcription efficiency by TAR-Tat system increases the functional expression of human olfactory receptors
Source: PLoS One. 2024 Jun 25;19(6):e0306029. doi: 10.1371/journal.pone.0306029 (PMC11198769; doi:10.1371/journal.pone.0306029)
Supplement: S2 Table — The term "n.d." stands for "not detected". (DOCX) [file pone.0306029.s002.docx]

S2 Table

| OR | hOR/pBApo | hOR/pHEK |
| --- | --- | --- |
| OR1A1 | n.d. | 995 |
| OR2B3 | n.d. | n.d. |
| OR2J3 | n.d. | 15 |
| OR2W1 | 21 | 60 |
| OR2Z1 | n.d. | n.d. |
| OR4K5 | n.d. | n.d. |
| OR5K1 | n.d. | 11 |
| OR5M3 | n.d. | n.d. |
| OR5P3 | 191 | 672 |
| OR8B3 | n.d. | n.d. |
| OR8B4 | n.d. | n.d. |
| OR8B8 | n.d. | n.d. |
| OR10A2 | n.d. | n.d. |
| OR10C1 | n.d. | n.d. |
| OR10H3 | n.d. | n.d. |
| OR52N4 | n.d. | n.d. |
| OR56B1 | n.d. | n.d. |
