# Supplementary figures and images for: Enhancement of transcription efficiency by TAR-Tat system increases the functional expression of human olfactory receptors
Source: PLoS One. 2024 Jun 25;19(6):e0306029. doi: 10.1371/journal.pone.0306029 (PMC11198769; doi:10.1371/journal.pone.0306029)

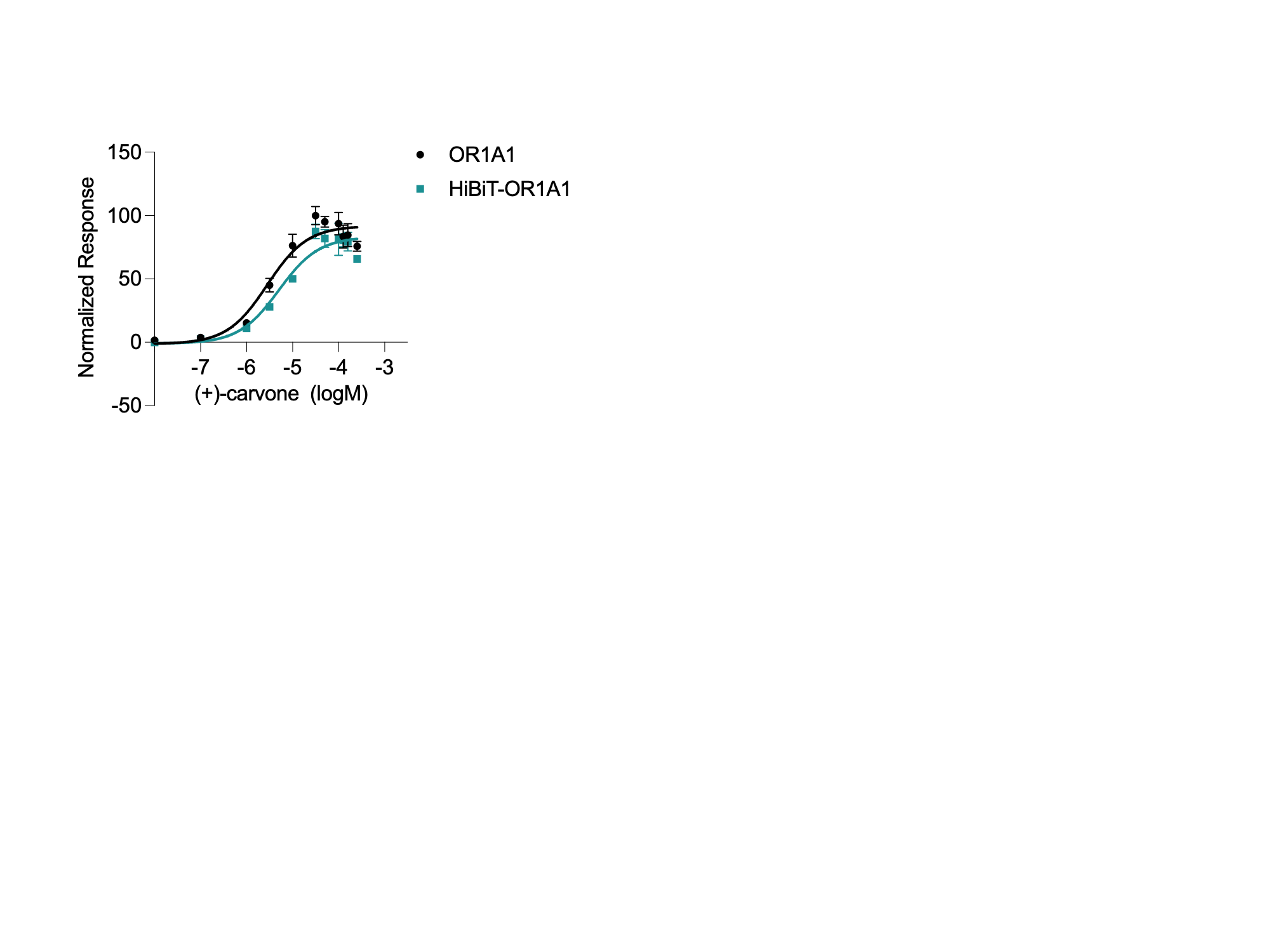

Supplement: S1 Fig — The responses of OR1A1 against (+)-carvone were evaluated with or without the HiBiT tag. The luminescence was normalized to a maximum value of 100. The y-axis denotes the normalized response, and the x-axis represents the concentration of (+)-carvone. Data are expressed as average (n = 3) ± SD (error bars). (TIF) [file pone.0306029.s003.tif]

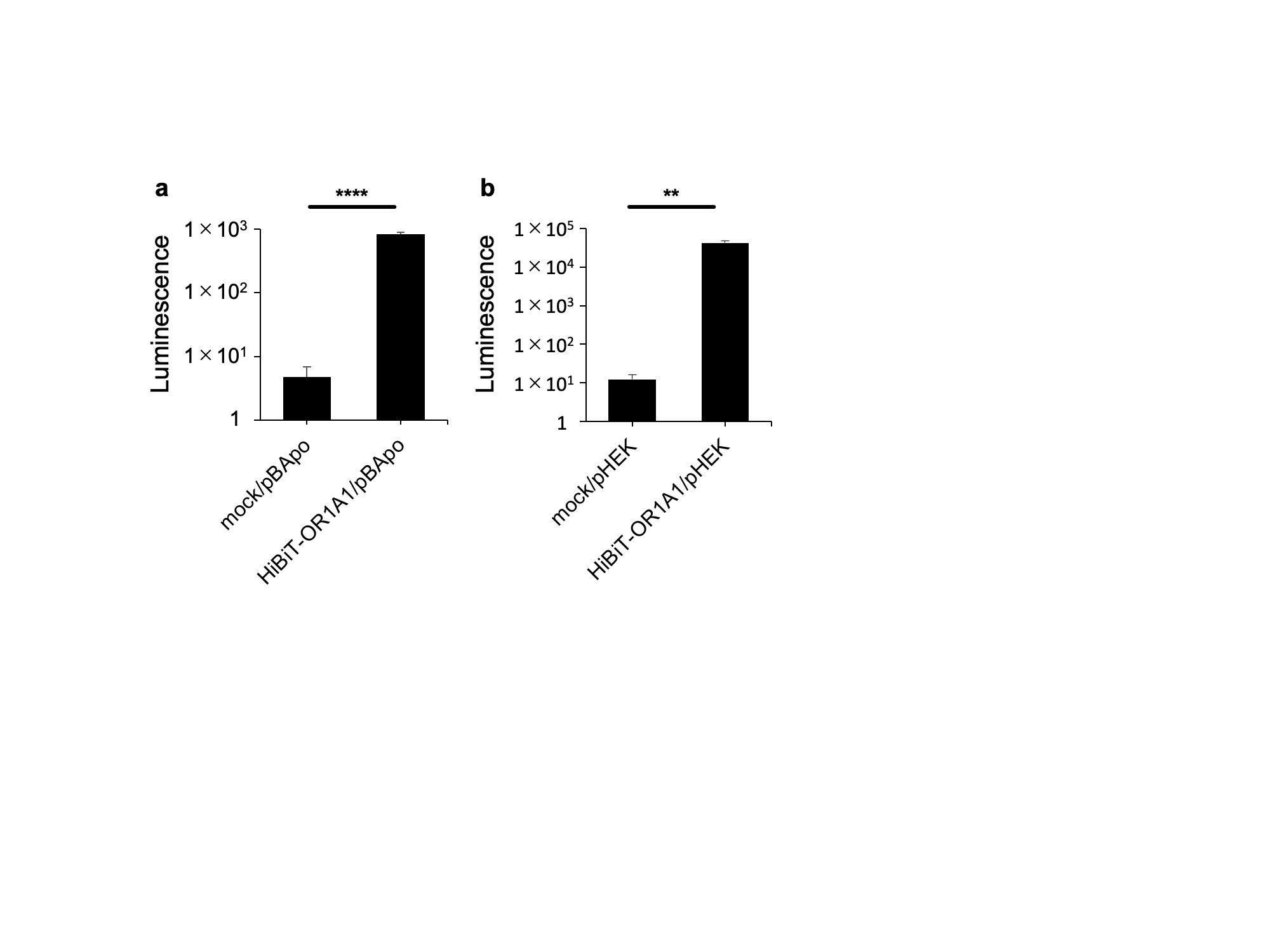

Supplement: S2 Fig — (TIF) [file pone.0306029.s004.tif]
